# Fasciite avec hyperéosinophile (syndrome de Shulman) chez une femme de 34 ans

**DOI:** 10.11604/pamj.2013.16.134.3573

**Published:** 2013-12-11

**Authors:** Faten Frikha, Zouhir Bahloul

**Affiliations:** 1 Service de Médecine Interne, CHU Hédi Chaker 3029, Sfax, Tunisie

**Keywords:** Fasciite, hyperéosinophile, syndrome de Shulman, fasciitis, hypereosinophilic, Shulman syndrome

## Image en médicine

Le syndrome de Shulman, appelé aussi fasciite avec hyperéosinophilie ou fasciite à éosinophiles, est une maladie rare qui associe sur le plan clinique une induration des tissus sous-cutanés plus ou moins étendue et sur le plan biologique une hyperéosinophilie sanguine. Nous rapportons l'observation d'une femme âgée de 34 ans qui était admise pour un syndrome oedémateux douloureux des 4 extrémités évoluant depuis 4 mois sans facteurs déclenchant. L'examen cutané objectivait un aspect infiltré luisant et scléreux de la peau des quatre membres s’étendant au tronc et épargnant le visage. Au niveau des avant-bras, la peau était indurée et d'aspect cartonné avec une dépression (vallée) selon le trajet veineux. A la biologie, la vitesse de sédimentation était à 127 mm à la 1ère heure. Il existait une hyperleucocytose avec une hyperéosinophilie à 7000 éléments/mm3. Le bilan immunologique avec recherche des AAN était négatif. Il n'y avait pas d'arguments pour une néoplasie ou une hémopathie sous-jacente. Le myélogramme était sans anomalies. Une biopsie cutanée profonde objectivait un derme fibrosé avec présence d'un infiltrat inflammatoire fait de lymphocytes, PNN et des histiocytes sans éosinophiles. Le diagnostic retenu était celui d'un syndrome de Shulman. Une corticothérapie à forte dose (prednisone 1 mg/Kg/jour) était instaurée ce qui a permis la régression complète de l'hyperéosinophilie (270 éléments/mm3). Cliniquement, on retrouvait une diminution nette de l'induration du derme des avant-bras et des membres inférieurs.

**Figure 1:**
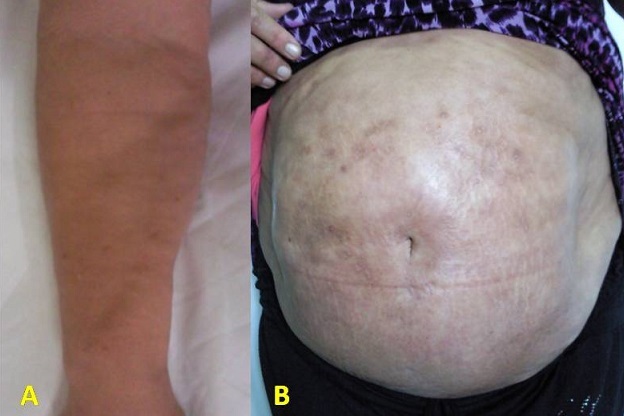
A) Sclérose de la peau de l'avant bras; B) Induration cutanée avec un aspect scléreux et cartonné du tronc

